# (*Z*)-3-(Anthracen-9-yl)-1-(2-eth­oxy­phen­yl)prop-2-en-1-one^1^

**DOI:** 10.1107/S1600536810038183

**Published:** 2010-09-30

**Authors:** Jaruwan Joothamongkhon, Suchada Chantrapromma, Thawanrat Kobkeatthawin, Hoong-Kun Fun

**Affiliations:** a Crystal Materials Research Unit, Department of Chemistry, Faculty of Science, Prince of Songkla University, Hat-Yai, Songkhla 90112, Thailand; b X-ray Crystallography Unit, School of Physics, Universiti Sains Malaysia, 11800 USM, Penang, Malaysia

## Abstract

The mol­ecule of the title chalcone, C_25_H_20_O_2_, consisting of 2-eth­oxy­phenyl and anthracene rings bridged by a prop-2-en-1-one unit, is twisted and exists in the *Z* configuration with respect to the central C=C bond. The dihedral angle between the benzene and anthracene rings is 78.17 (9)°. The propene unit makes dihedral angles of 44.5 (2) and 81.1 (2)° with the benzene and anthracene rings, respectively. The eth­oxy substituent is almost coplanar with the attached benzene ring [C—O—C—C torsion angle = 178.57 (19)°]. In the crystal, mol­ecules are linked into chains along the *a* axis by weak C—H⋯O inter­actions. The crystal structure is further stabilized by C—H⋯π inter­actions.

## Related literature

For bond-length data, see: Allen *et al.* (1987 ▶). For related structures, see: Chantrapromma *et al.* (2009 ▶, 2010 ▶); Suwunwong *et al.* (2009 ▶). For background to and applications of chalcones, see: Kobkeatthawin *et al.* (2010 ▶); Nowakowska (2007 ▶); Oliveira *et al.* (2007 ▶); Patil & Dharmaprakash (2008 ▶); Saydam *et al.* (2003 ▶); Svetlichny *et al.* (2007 ▶); Tewtrakul *et al.* (2003 ▶). For the stability of the temperature controller used in the data collection, see Cosier & Glazer, (1986 ▶).
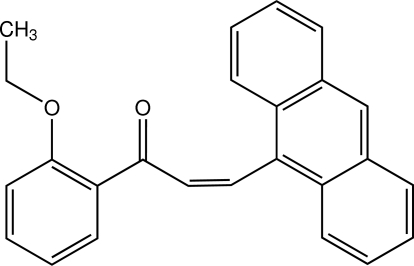


## Experimental

### 

#### Crystal data

C_25_H_20_O_2_
*M* *_r_* = 352.41
Orthorhombic, 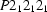

*a* = 5.4442 (1) Å
*b* = 10.7665 (2) Å
*c* = 32.2160 (7) Å
*V* = 1888.34 (6) Å^3^
*Z* = 4
Mo *K*α radiation
μ = 0.08 mm^−1^
*T* = 100 K
0.47 × 0.16 × 0.07 mm

#### Data collection

Bruker APEXII CCD area-detector diffractometer
Absorption correction: multi-scan (*SADABS*; Bruker, 2005 ▶) *T*_min_ = 0.964, *T*_max_ = 0.995
18936 measured reflections
3190 independent reflections
2632 reflections with *I* > 2σ(*I*)
*R*_int_ = 0.055

#### Refinement

*R*[*F* ^2^ > 2σ(*F* ^2^)] = 0.044
*wR*(*F* ^2^) = 0.102
*S* = 1.04
3190 reflections
245 parameters
H-atom parameters constrained
Δρ_max_ = 0.29 e Å^−3^
Δρ_min_ = −0.19 e Å^−3^

### 

Data collection: *APEX2* (Bruker, 2005 ▶); cell refinement: *SAINT* (Bruker, 2005 ▶); data reduction: *SAINT*; program(s) used to solve structure: *SHELXTL* (Sheldrick, 2008 ▶); program(s) used to refine structure: *SHELXTL*; molecular graphics: *SHELXTL*; software used to prepare material for publication: *SHELXTL* and *PLATON* (Spek, 2009 ▶).

## Supplementary Material

Crystal structure: contains datablocks global, I. DOI: 10.1107/S1600536810038183/rz2490sup1.cif

Structure factors: contains datablocks I. DOI: 10.1107/S1600536810038183/rz2490Isup2.hkl

Additional supplementary materials:  crystallographic information; 3D view; checkCIF report

## Figures and Tables

**Table 1:** Hydrogen-bond geometry (Å, °) *Cg*1 and *Cg*2 are the centroids of the C1–C6 and C10–C11/C16–C18/C23 rings, respectively.

*D*—H⋯*A*	*D*—H	H⋯*A*	*D*⋯*A*	*D*—H⋯*A*
C3—H3*A*⋯O1^i^	0.93	2.59	3.205 (3)	124
C8—H8*A*⋯O1^ii^	0.93	2.35	3.093 (2)	136
C9—H9*A*⋯*Cg*2^ii^	0.93	2.88	3.7609 (19)	160
C24—H24*A*⋯*Cg*1^ii^	0.97	2.86	3.739 (2)	151

Symmetry codes: (i) 
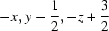
; (ii) 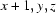.

**Fig. 1.**
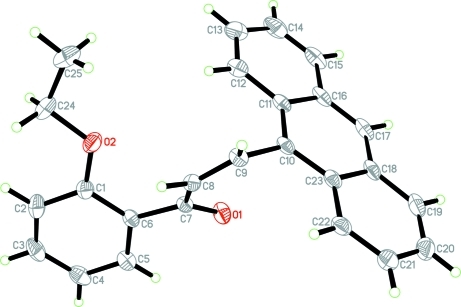
The molecular structure of the title compound, showing 50% probability displacement ellipsoids and the atom-numbering scheme.

**Fig. 2.**
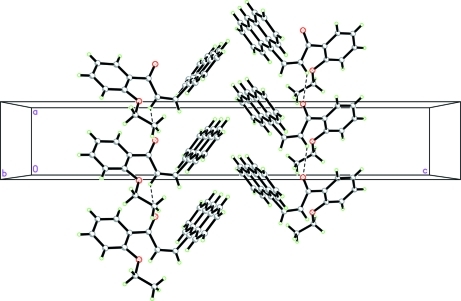
The crystal packing of the title compound viewed along the b axis, showing chains running along the a axis. C—H···O weak interactions are shown as dashed lines.

## supplementary crystallographic information

### Comment

Chalcones are an interesting class of compounds which have been reported to posses various useful properties. They have been studied for non-linear optical (Patil & Dharmaprakash, 2008), biological activities including anti-inflammatory, antileishmanial, antimicrobial, antioxidant (Nowakowska, 2007; Oliveira *et al.*, 2007; Saydam *et al.*, 2003) and HIV-1 protease inhibitory (Tewtrakul *et al.*, 2003) as well as fluorescence properties (Kobkeatthawin *et al.*, 2010; Svetlichny *et al.*, 2007). We have previously reported the crystal structures of several chalcone derivatives containing the anthracene moiety which show interesting fluorescence properties (Suwunwong *et al.*, 2009; Chantrapromma *et al.*, 2009, 2010). Due to its various interesting properties the title chalcone derivative (I) was synthesized in order to study its NLO and fluorescence properties. The title compound crystallizes in the orthorhombic noncentrosymmetric space group *P*2_1_2_1_2_1_ and therefore it should exhibit second-order non-linear optic properties. In addition our experiment shows that (I) has fluorescence property. Herein the crystal structure of (I) is reported.

The molecule of (I) (Fig. 1) exists in an *Z* configuration respected to the C8═C9 double bond [1.331 (3)°] and the C7–C8–C9–C10 torsion angle is 4.2 (3)°. The anthracene unit is essentially planar with the maximum deviation of -0.049 (2) Å for atom C10 [*r.m.s*. 0.0125 (2) Å]. The total molecule is twisted with the dihedral angle between benzene and anthracene rings of 78.17 (9)°. The mean plane through the propene unit (C7–C9) makes dihedral angles of 44.5 (2) and 81.1 (2)° with the benzene and anthracene rings, respectively. Atom O1 of the pro-2-en-1-one moiety is deviated from the propene plane as indicated by the torsion angle O1–C7–C8–C9 = 19.0 (3)°. The ethoxy substituent is coplanar with the attached benzene ring with the torsion angle C1–O2–C24–C25 = 178.57 (19)°. The bond distances are of normal values (Allen *et al.*, 1987) and are comparable with those found in related structures (Chantrapromma *et al.*, 2009; 2010; Suwunwong *et al.*, 2009).

In the crystal packing, the molecules are linked into chains along the *a* axis through the prop-2-en-1-one unit by weak C—H···O interactions (Fig. 2). The crystal structure is further stabilized by C—H···π interactions (Table 1); *Cg*_1_ and *Cg*_2_ are the centroids of the C1–C6 and C10–C11/C16–C18/C23 rings, respectively.

### Experimental

The title compound was synthesized by the condensation of anthracene-9-carbaldehyde (2 mmol, 0.41 g) with 2-ethoxyacetophenone (2 mmol, 0.33 g) in ethanol (40 ml) in the presence of NaOH(aq) (5 ml, 20%). After stirring for 4 hr at room temperature, a yellow solid appeared and was then collected by filtration, washed with distilled water and dried in air. Yellow plate-shaped single crystals of the title compound suitable for *X*-ray structure determination were recrystalized from methanol by slow evaporation of the solvent at room temperature after several days, Mp. 419–421 K.

### Refinement

All H atoms were positioned geometrically and allowed to ride on their parent atoms, with d(C—H) = 0.93 Å for aromatic and CH, 0.97 Å for CH_2_ and 0.96 Å for CH_3_ atoms. The *U*_iso_ values were constrained to be 1.5*U*_eq_ of the carrier atom for methyl H atoms and 1.2*U*_eq_ for the remaining H atoms. A rotating group model was used for the methyl groups. The highest residual electron density peak is located at 0.80Å from atom C8 and the deepest hole is located at 0.76 Å from atom C10. A total of 2321 Friedel pairs were merged before final refinement as there is no large anomalous dispersion for the determination of the absolute configuration.

### Crystal data

**Table:**

C_25_H_20_O_2_	*D*_x_ = 1.240 Mg m^−^^3^
*M**_r_* = 352.41	Melting point = 419–421 K
Orthorhombic, *P*2_1_2_1_2_1_	Mo *K*α radiation, λ = 0.71073 Å
Hall symbol: P 2ac 2ab	Cell parameters from 3190 reflections
*a* = 5.4442 (1) Å	θ = 2.0–30.0°
*b* = 10.7665 (2) Å	µ = 0.08 mm^−^^1^
*c* = 32.2160 (7) Å	*T* = 100 K
*V* = 1888.34 (6) Å^3^	Plate, yellow
*Z* = 4	0.47 × 0.16 × 0.07 mm
*F*(000) = 744	

### Data collection

**Table:**

Bruker APEXII CCD area-detector diffractometer	3190 independent reflections
Radiation source: sealed tube	2632 reflections with *I* > 2σ(*I*)
graphite	*R*_int_ = 0.055
φ and ω scans	θ_max_ = 30.0°, θ_min_ = 2.0°
Absorption correction: multi-scan (*SADABS*; Bruker, 2005)	*h* = −7→7
*T*_min_ = 0.964, *T*_max_ = 0.995	*k* = −13→15
18936 measured reflections	*l* = −38→45

### Refinement

**Table:**

Refinement on *F*^2^	Primary atom site location: structure-invariant direct methods
Least-squares matrix: full	Secondary atom site location: difference Fourier map
*R*[*F*^2^ > 2σ(*F*^2^)] = 0.044	Hydrogen site location: inferred from neighbouring sites
*wR*(*F*^2^) = 0.102	H-atom parameters constrained
*S* = 1.03	*w* = 1/[σ^2^(*F*_o_^2^) + (0.042*P*)^2^ + 0.4942*P*] where *P* = (*F*_o_^2^ + 2*F*_c_^2^)/3
3190 reflections	(Δ/σ)_max_ = 0.001
245 parameters	Δρ_max_ = 0.29 e Å^−^^3^
0 restraints	Δρ_min_ = −0.19 e Å^−^^3^

### Special details

**Table:**

Experimental. The crystal was placed in the cold stream of an Oxford Cryosystems Cobra open-flow nitrogen cryostat (Cosier & Glazer, 1986) operating at 120.0 (1) K.
Geometry. All e.s.d.'s (except the e.s.d. in the dihedral angle between two l.s. planes) are estimated using the full covariance matrix. The cell e.s.d.'s are taken into account individually in the estimation of e.s.d.'s in distances, angles and torsion angles; correlations between e.s.d.'s in cell parameters are only used when they are defined by crystal symmetry. An approximate (isotropic) treatment of cell e.s.d.'s is used for estimating e.s.d.'s involving l.s. planes.
Refinement. Refinement of *F*^2^ against ALL reflections. The weighted *R*-factor *wR* and goodness of fit *S* are based on *F*^2^, conventional *R*-factors *R* are based on *F*, with *F* set to zero for negative *F*^2^. The threshold expression of *F*^2^ > σ(*F*^2^) is used only for calculating *R*-factors(gt) *etc*. and is not relevant to the choice of reflections for refinement. *R*-factors based on *F*^2^ are statistically about twice as large as those based on *F*, and *R*- factors based on ALL data will be even larger.

### Fractional atomic coordinates and isotropic or equivalent isotropic displacement parameters (Å^2^)

**Table:**

	*x*	*y*	*z*	*U*_iso_*/*U*_eq_	
O1	−0.0044 (2)	0.12896 (14)	0.83325 (4)	0.0251 (3)	
O2	0.5039 (3)	−0.11326 (13)	0.80475 (4)	0.0281 (3)	
C1	0.3555 (4)	−0.08224 (18)	0.77249 (6)	0.0222 (4)	
C2	0.3530 (4)	−0.1433 (2)	0.73429 (6)	0.0290 (5)	
H2A	0.4665	−0.2057	0.7288	0.035*	
C3	0.1806 (4)	−0.1105 (2)	0.70459 (6)	0.0311 (5)	
H3A	0.1805	−0.1508	0.6791	0.037*	
C4	0.0084 (4)	−0.0187 (2)	0.71237 (6)	0.0290 (4)	
H4A	−0.1093	0.0013	0.6925	0.035*	
C5	0.0135 (4)	0.04316 (19)	0.75010 (6)	0.0242 (4)	
H5A	−0.1019	0.1048	0.7554	0.029*	
C6	0.1884 (3)	0.01477 (17)	0.78025 (5)	0.0187 (4)	
C7	0.1887 (3)	0.08877 (17)	0.81949 (6)	0.0174 (4)	
C8	0.4287 (3)	0.11964 (17)	0.83872 (6)	0.0175 (4)	
H8A	0.5698	0.1084	0.8229	0.021*	
C9	0.4541 (3)	0.16241 (17)	0.87726 (6)	0.0193 (4)	
H9A	0.6114	0.1843	0.8858	0.023*	
C10	0.2511 (3)	0.17797 (19)	0.90773 (5)	0.0194 (4)	
C11	0.1582 (3)	0.07403 (18)	0.92908 (6)	0.0207 (4)	
C12	0.2619 (4)	−0.04731 (19)	0.92423 (6)	0.0261 (4)	
H12A	0.3957	−0.0585	0.9067	0.031*	
C13	0.1672 (5)	−0.1464 (2)	0.94501 (7)	0.0326 (5)	
H13A	0.2386	−0.2243	0.9419	0.039*	
C14	−0.0399 (5)	−0.1318 (2)	0.97134 (7)	0.0352 (5)	
H14A	−0.1049	−0.2006	0.9849	0.042*	
C15	−0.1435 (4)	−0.0192 (2)	0.97689 (6)	0.0317 (5)	
H15A	−0.2787	−0.0115	0.9943	0.038*	
C16	−0.0488 (4)	0.0885 (2)	0.95640 (6)	0.0244 (4)	
C17	−0.1518 (4)	0.2059 (2)	0.96189 (6)	0.0264 (4)	
H17A	−0.2869	0.2147	0.9793	0.032*	
C18	−0.0574 (3)	0.3102 (2)	0.94196 (6)	0.0239 (4)	
C19	−0.1629 (4)	0.4308 (2)	0.94695 (6)	0.0304 (5)	
H19A	−0.2990	0.4406	0.9641	0.036*	
C20	−0.0687 (4)	0.5313 (2)	0.92721 (7)	0.0337 (5)	
H20A	−0.1417	0.6087	0.9306	0.040*	
C21	0.1408 (4)	0.5188 (2)	0.90134 (6)	0.0301 (5)	
H21A	0.2072	0.5887	0.8886	0.036*	
C22	0.2456 (4)	0.40556 (19)	0.89496 (6)	0.0243 (4)	
H22A	0.3804	0.3988	0.8774	0.029*	
C23	0.1515 (3)	0.29688 (19)	0.91489 (6)	0.0211 (4)	
C24	0.6799 (4)	−0.2118 (2)	0.79972 (7)	0.0326 (5)	
H24A	0.7919	−0.1932	0.7771	0.039*	
H24C	0.5973	−0.2896	0.7937	0.039*	
C25	0.8164 (5)	−0.2200 (2)	0.84010 (8)	0.0457 (6)	
H25C	0.9491	−0.2782	0.8374	0.069*	
H25A	0.7066	−0.2473	0.8616	0.069*	
H25B	0.8810	−0.1397	0.8472	0.069*	

### Atomic displacement parameters (Å^2^)

**Table:**

	*U*^11^	*U*^22^	*U*^33^	*U*^12^	*U*^13^	*U*^23^
O1	0.0146 (5)	0.0381 (8)	0.0226 (7)	0.0038 (6)	−0.0007 (5)	−0.0056 (6)
O2	0.0291 (7)	0.0251 (7)	0.0299 (7)	0.0092 (6)	−0.0045 (6)	−0.0046 (6)
C1	0.0216 (8)	0.0224 (9)	0.0226 (9)	−0.0010 (7)	0.0011 (8)	−0.0015 (8)
C2	0.0299 (10)	0.0262 (10)	0.0310 (11)	−0.0021 (9)	0.0050 (9)	−0.0109 (9)
C3	0.0395 (11)	0.0340 (12)	0.0199 (10)	−0.0130 (10)	0.0033 (9)	−0.0074 (9)
C4	0.0300 (10)	0.0369 (11)	0.0200 (9)	−0.0085 (10)	−0.0058 (8)	0.0006 (9)
C5	0.0207 (8)	0.0299 (10)	0.0221 (9)	−0.0045 (8)	−0.0010 (8)	−0.0010 (8)
C6	0.0161 (8)	0.0231 (9)	0.0170 (8)	−0.0028 (7)	0.0015 (7)	−0.0006 (7)
C7	0.0150 (7)	0.0214 (9)	0.0160 (8)	0.0003 (7)	−0.0002 (7)	0.0013 (7)
C8	0.0119 (7)	0.0207 (9)	0.0198 (9)	0.0012 (6)	0.0013 (6)	0.0015 (8)
C9	0.0127 (7)	0.0260 (9)	0.0190 (9)	−0.0005 (7)	−0.0004 (7)	0.0010 (7)
C10	0.0157 (7)	0.0304 (10)	0.0122 (8)	0.0002 (7)	−0.0028 (6)	−0.0014 (8)
C11	0.0188 (8)	0.0299 (10)	0.0133 (8)	−0.0035 (8)	−0.0024 (7)	−0.0004 (7)
C12	0.0255 (9)	0.0312 (11)	0.0216 (10)	−0.0014 (8)	−0.0018 (8)	0.0027 (8)
C13	0.0406 (12)	0.0299 (11)	0.0272 (11)	−0.0053 (10)	−0.0069 (10)	0.0046 (9)
C14	0.0441 (13)	0.0390 (13)	0.0226 (10)	−0.0184 (11)	0.0001 (10)	0.0055 (9)
C15	0.0301 (10)	0.0476 (13)	0.0173 (9)	−0.0124 (10)	0.0012 (9)	0.0003 (9)
C16	0.0213 (9)	0.0395 (12)	0.0123 (8)	−0.0058 (8)	−0.0012 (7)	−0.0020 (8)
C17	0.0181 (8)	0.0472 (13)	0.0138 (8)	−0.0011 (9)	0.0001 (7)	−0.0051 (9)
C18	0.0199 (8)	0.0377 (11)	0.0141 (8)	0.0039 (8)	−0.0037 (7)	−0.0066 (8)
C19	0.0267 (10)	0.0450 (13)	0.0195 (10)	0.0115 (10)	−0.0039 (8)	−0.0102 (9)
C20	0.0374 (11)	0.0362 (12)	0.0275 (11)	0.0151 (10)	−0.0078 (9)	−0.0084 (10)
C21	0.0365 (11)	0.0303 (11)	0.0234 (10)	0.0042 (10)	−0.0066 (9)	−0.0016 (9)
C22	0.0256 (9)	0.0307 (11)	0.0165 (9)	0.0025 (8)	−0.0031 (7)	0.0003 (8)
C23	0.0186 (8)	0.0304 (10)	0.0143 (8)	0.0012 (8)	−0.0047 (7)	−0.0026 (7)
C24	0.0318 (10)	0.0232 (10)	0.0427 (13)	0.0091 (9)	0.0012 (10)	−0.0019 (9)
C25	0.0477 (14)	0.0398 (14)	0.0496 (15)	0.0210 (12)	−0.0065 (13)	0.0051 (12)

### Geometric parameters (Å, °)

**Table:**

O1—C7	1.220 (2)	C13—C14	1.420 (3)
O2—C1	1.358 (2)	C13—H13A	0.9300
O2—C24	1.438 (2)	C14—C15	1.349 (3)
C1—C2	1.395 (3)	C14—H14A	0.9300
C1—C6	1.407 (3)	C15—C16	1.430 (3)
C2—C3	1.386 (3)	C15—H15A	0.9300
C2—H2A	0.9300	C16—C17	1.394 (3)
C3—C4	1.386 (3)	C17—C18	1.392 (3)
C3—H3A	0.9300	C17—H17A	0.9300
C4—C5	1.386 (3)	C18—C19	1.429 (3)
C4—H4A	0.9300	C18—C23	1.440 (3)
C5—C6	1.394 (3)	C19—C20	1.356 (3)
C5—H5A	0.9300	C19—H19A	0.9300
C6—C7	1.494 (3)	C20—C21	1.419 (3)
C7—C8	1.483 (2)	C20—H20A	0.9300
C8—C9	1.331 (3)	C21—C22	1.362 (3)
C8—H8A	0.9300	C21—H21A	0.9300
C9—C10	1.488 (2)	C22—C23	1.430 (3)
C9—H9A	0.9300	C22—H22A	0.9300
C10—C11	1.408 (3)	C24—C25	1.501 (3)
C10—C23	1.409 (3)	C24—H24A	0.9700
C11—C12	1.432 (3)	C24—H24C	0.9700
C11—C16	1.438 (3)	C25—H25C	0.9600
C12—C13	1.361 (3)	C25—H25A	0.9600
C12—H12A	0.9300	C25—H25B	0.9600
			
C1—O2—C24	119.44 (16)	C15—C14—H14A	119.6
O2—C1—C2	124.38 (18)	C13—C14—H14A	119.6
O2—C1—C6	115.55 (16)	C14—C15—C16	121.1 (2)
C2—C1—C6	120.01 (18)	C14—C15—H15A	119.4
C3—C2—C1	119.7 (2)	C16—C15—H15A	119.4
C3—C2—H2A	120.2	C17—C16—C15	122.11 (18)
C1—C2—H2A	120.2	C17—C16—C11	119.38 (19)
C4—C3—C2	121.02 (19)	C15—C16—C11	118.5 (2)
C4—C3—H3A	119.5	C18—C17—C16	121.65 (18)
C2—C3—H3A	119.5	C18—C17—H17A	119.2
C5—C4—C3	119.2 (2)	C16—C17—H17A	119.2
C5—C4—H4A	120.4	C17—C18—C19	122.20 (18)
C3—C4—H4A	120.4	C17—C18—C23	119.37 (19)
C4—C5—C6	121.3 (2)	C19—C18—C23	118.42 (19)
C4—C5—H5A	119.3	C20—C19—C18	121.36 (19)
C6—C5—H5A	119.3	C20—C19—H19A	119.3
C5—C6—C1	118.72 (17)	C18—C19—H19A	119.3
C5—C6—C7	118.25 (16)	C19—C20—C21	120.3 (2)
C1—C6—C7	123.03 (16)	C19—C20—H20A	119.9
O1—C7—C8	121.85 (16)	C21—C20—H20A	119.9
O1—C7—C6	119.69 (16)	C22—C21—C20	120.7 (2)
C8—C7—C6	118.27 (15)	C22—C21—H21A	119.7
C9—C8—C7	123.95 (16)	C20—C21—H21A	119.7
C9—C8—H8A	118.0	C21—C22—C23	120.98 (19)
C7—C8—H8A	118.0	C21—C22—H22A	119.5
C8—C9—C10	125.25 (16)	C23—C22—H22A	119.5
C8—C9—H9A	117.4	C10—C23—C22	122.13 (17)
C10—C9—H9A	117.4	C10—C23—C18	119.57 (18)
C11—C10—C23	120.25 (16)	C22—C23—C18	118.26 (18)
C11—C10—C9	119.99 (17)	O2—C24—C25	106.01 (18)
C23—C10—C9	119.76 (17)	O2—C24—H24A	110.5
C10—C11—C12	122.03 (17)	C25—C24—H24A	110.5
C10—C11—C16	119.65 (18)	O2—C24—H24C	110.5
C12—C11—C16	118.32 (18)	C25—C24—H24C	110.5
C13—C12—C11	120.80 (19)	H24A—C24—H24C	108.7
C13—C12—H12A	119.6	C24—C25—H25C	109.5
C11—C12—H12A	119.6	C24—C25—H25A	109.5
C12—C13—C14	120.5 (2)	H25C—C25—H25A	109.5
C12—C13—H13A	119.7	C24—C25—H25B	109.5
C14—C13—H13A	119.7	H25C—C25—H25B	109.5
C15—C14—C13	120.7 (2)	H25A—C25—H25B	109.5
			
C24—O2—C1—C2	2.8 (3)	C12—C13—C14—C15	−1.3 (3)
C24—O2—C1—C6	179.74 (18)	C13—C14—C15—C16	0.2 (3)
O2—C1—C2—C3	174.88 (19)	C14—C15—C16—C17	−179.5 (2)
C6—C1—C2—C3	−2.0 (3)	C14—C15—C16—C11	0.9 (3)
C1—C2—C3—C4	−0.7 (3)	C10—C11—C16—C17	−1.1 (3)
C2—C3—C4—C5	1.6 (3)	C12—C11—C16—C17	179.48 (18)
C3—C4—C5—C6	0.0 (3)	C10—C11—C16—C15	178.43 (17)
C4—C5—C6—C1	−2.6 (3)	C12—C11—C16—C15	−0.9 (3)
C4—C5—C6—C7	177.64 (18)	C15—C16—C17—C18	179.67 (18)
O2—C1—C6—C5	−173.54 (17)	C11—C16—C17—C18	−0.8 (3)
C2—C1—C6—C5	3.6 (3)	C16—C17—C18—C19	179.27 (18)
O2—C1—C6—C7	6.2 (3)	C16—C17—C18—C23	0.1 (3)
C2—C1—C6—C7	−176.71 (18)	C17—C18—C19—C20	−179.9 (2)
C5—C6—C7—O1	32.2 (3)	C23—C18—C19—C20	−0.7 (3)
C1—C6—C7—O1	−147.54 (19)	C18—C19—C20—C21	−0.8 (3)
C5—C6—C7—C8	−142.93 (18)	C19—C20—C21—C22	2.0 (3)
C1—C6—C7—C8	37.4 (3)	C20—C21—C22—C23	−1.5 (3)
O1—C7—C8—C9	19.0 (3)	C11—C10—C23—C22	177.84 (18)
C6—C7—C8—C9	−166.01 (18)	C9—C10—C23—C22	−2.6 (3)
C7—C8—C9—C10	4.2 (3)	C11—C10—C23—C18	−4.4 (3)
C8—C9—C10—C11	78.2 (2)	C9—C10—C23—C18	175.19 (16)
C8—C9—C10—C23	−101.4 (2)	C21—C22—C23—C10	177.78 (18)
C23—C10—C11—C12	−176.92 (18)	C21—C22—C23—C18	0.0 (3)
C9—C10—C11—C12	3.5 (3)	C17—C18—C23—C10	2.5 (3)
C23—C10—C11—C16	3.7 (3)	C19—C18—C23—C10	−176.73 (17)
C9—C10—C11—C16	−175.84 (16)	C17—C18—C23—C22	−179.67 (18)
C10—C11—C12—C13	−179.47 (19)	C19—C18—C23—C22	1.1 (3)
C16—C11—C12—C13	−0.1 (3)	C1—O2—C24—C25	178.57 (19)
C11—C12—C13—C14	1.2 (3)		

### Hydrogen-bond geometry (Å, °)

**Table:**

*Cg*1 and *Cg*2 are the centroids of the C1–C6 and C10–C11/C16–C18/C23 rings, respectively.

**Table:**

*D*—H···*A*	*D*—H	H···*A*	*D*···*A*	*D*—H···*A*
C3—H3A···O1^i^	0.93	2.59	3.205 (3)	124
C8—H8A···O1^ii^	0.93	2.35	3.093 (2)	136
C9—H9A···Cg2^ii^	0.93	2.88	3.7609 (19)	160
C24—H24A···Cg1^ii^	0.97	2.86	3.739 (2)	151

Symmetry codes: (i) −*x*, *y*−1/2, −*z*+3/2; (ii) *x*+1, *y*, *z*.
